# Analysis of the drug target of the anti-tuberculosis compound OCT313: phosphotransacetylase is a potential drug target for anti-mycobacterial agents

**DOI:** 10.1128/msphere.00463-25

**Published:** 2025-11-28

**Authors:** Takemasa Takii, Tomohiro Hasegawa, Saotomo Itoh, Shinji Maeda, Takayuki Wada, Yasuhiro Horita, Akihito Nishiyama, Sohkichi Matsumoto, Naoya Ohara, Aoi Kimishima, Yukihiro Asami, Shigeaki Hida, Kikuo Onozaki

**Affiliations:** 1 Department of Mycobacterium Reference and Research, the Research Institute of Tuberculosis, Japan Anti-Tuberculosis Association 46635 , Kiyose, Tokyo, Japan; 2 Department of Hygienic Chemistry, Graduate School of Pharmaceutical Sciences, Nagoya City University 12963, Nagoya, Aichi, Japan; 3 Ōmura Satoshi Memorial Institute, Kitasato University 12877, Minato-ku, Tokyo, Japan; 4 Laboratory of Hygienic Chemistry, School of Pharmacy, Aichi Gakuin University 57898, Nagoya, Aichi, Japan; 5 Graduate School of Pharmaceutical Sciences, Hokkaido University of Sciences, Sapporo, Hokkaido, Japan; 6 Department of Microbiology, Graduate School of Human Life and Ecology, Osaka Metropolitan University 12936, Osaka, Japan; 7 Osaka International Research Center for Infectious Diseases, Osaka Metropolitan University, Osaka, Osaka, Japan; 8 Department of Clinical Pharmaceutics, Graduate School of Medical Sciences, Nagoya City University 12963, Nagoya, Aichi, Japan; 9 Department of Bacteriology, Niigata University School of Medicine 12978, Niigata, Japan; 10 Department of Bacteriology, Osaka Metropolitan University Graduate School of Medicine 12936, Osaka, Japan; 11 Division of Research Aids, Hokkaido University Institute for Vaccine Research & Development 12810, Sapporo, Hokkaido, Japan; 12 Laboratory of Tuberculosis, Institute of Tropical Disease, Universitas Airlangga 148005, Surabaya, Indonesia; 13 Department of Oral Microbiology, Graduate School of Medicine, Dentistry and Pharmaceutical Sciences, Okayama University 12997, Okayama, Japan; 14 Laboratory of Applied Microbial Chemistry, Ōmura Satoshi Memorial Institute, Kitasato University 12877, Minato-ku, Tokyo, Japan; Medical College of Wisconsin, Milwaukee, Wisconsin, USA

**Keywords:** phosphotransacetylase, acetyl coenzyme A, dithiocarbamate, *N*-acetyl glucosamine, anti-mycobacterial agents, latent tuberculosis infection

## Abstract

Tuberculosis (TB) is one of the most common infectious diseases caused by bacteria worldwide. The increasing prevalence of multidrug-resistant TB (MDR-TB) and latent TB infection (LTBI) has intensified the global TB burden. Therefore, the development of new drugs for MDR-TB and LTBI is urgently required. We have reported that the derivative of dithiocarbamate sugar derivative, 2-acetamido-2-deoxy-β-D-glucopyranosyl *N,N*-dimethyldithiocarbamate (OCT313), exhibits anti-mycobacterial activity against MDR-MTB. Here, we identified the target of OCT313. In experimentally generated OCT313-resistant bacteria, adenine at position 1,092 in the metabolic enzyme phosphotransacetylase (PTA) gene was replaced with cytosine. This mutation is a nonsynonymous mutation that converts methionine to leucine at position 365 in the PTA protein. OCT313 inhibited the enzymatic activity of recombinant wild-type PTA, but not of the mutant PTA (M365L). PTA is an enzyme that produces acetyl-coenzyme A (acetyl-CoA) from acetyl phosphate and CoA and is involved in metabolic pathways; therefore, it was expected to also be active against dormant *Mycobacterium tuberculosis* bacilli. OCT313 exhibits antibacterial activity in the Wayne model of dormancy using *Mycobacterium bovis* BCG, and overexpression of PTA in OCT313-resistant bacilli restored sensitivity to OCT313. Collectively, the target of OCT313 is PTA, and OCT313 is a promising antimicrobial candidate for MDR-TB and LTBI.

**IMPORTANCE:**

Through this study, we propose a new target for the development of medicines to treat multidrug-resistant tuberculosis and latent tuberculosis infection. The target enzyme phosphotransacetylase (PTA) is a key enzyme that functions in major metabolic pathways, and the homologous structures of PTA enzymes vary greatly among bacterial species. Since the treatment of mycobacterial disease is long term, the development of antibiotics targeting PTA is useful for species-specific therapy.

## INTRODUCTION

Tuberculosis (TB) remains one of the world’s largest infectious diseases. More than 10 million people fall ill with this disease every year, and the number has been rising since 2021 owing to the coronavirus disease 2019 pandemic (1). The World Health Organization (WHO) estimates that 10.8 million new cases of TB and 1.25 million deaths worldwide in 2023, including 1.09 million among human immunodeficiency virus (HIV)-negative people and 161,000 among people with HIV (1). The WHO standard treatment regimen for TB consists of four drugs: isoniazid (INH), rifampicin (RFP), pyrazinamide, and ethambutol (EB) taken for 2 months, followed by two more drugs, INH and RFP, for another 4 months (1). Due to various issues, such as medication compliance, drug-resistant *Mycobacterium tuberculosis* bacilli are on the rise, and cases of initial infection with these drug-resistant *M. tuberculosis* bacilli are occurring. In particular, multidrug-resistant TB (MDR-TB) that is resistant to INH and RFP has significant antibacterial activity, is increasing, and the development of new drugs is desired. Bedaquiline and delamanid are the first new anti-TB drugs developed in nearly 50 years that show efficacy against MDR-TB (2). These drugs are believed to act through the inhibition of ATP synthase (3) and mycolic acid synthesis (4). Resistant TB has already been reported with these new drugs (5, 6), and there is a need to develop new drugs with novel modes of action. Furthermore, not all infected people develop TB; many remain in a state of latent TB infection (LTBI), but those with HIV infection or immunocompromised individuals are at increased risk of developing the disease. There is also a need to develop bactericidal drugs against dormant *M. tuberculosis* bacilli and LTBI.

In a previous study, we screened a compound library of sugar derivatives and found that 2-acetamido-2-deoxy-β-D-glucopyranosyl *N,N*-dimethyldithiocarbamate (OCT313) exhibited anti-TB activity (7) (Fig. 8). OCT313, a dithiocarbamate sugar derivative, and OCT313HK (8), a sugar derivative in which the dithiocarbamate was replaced with a pyrrolidine skeleton, exhibiting antibacterial activity against MDR *M. tuberculosis* bacilli. The structure-activity relationship of these sugar derivatives revealed that the active functional group is dithiocarbamate. Furthermore, we have reported that disulfiram (DSF), an analog of the active functional group, exhibits antibacterial activity and has therapeutic effects in a mouse model of chronic TB infection (9). In this study, we sought to analyze its site of action.

## MATERIALS AND METHODS

### Reagents

Phosphotransacetylase (PTA) from *Leuconostoc mesenteroides* and *N*-ethylmaleimide (NEM) were purchased from Wako Pure Chemical Industries, Ltd., Osaka, Japan. OCT313 (Fig. 8) was provided by Dr. Taku Chiba (Nagoya City University). Dimethyldithiocarbamate (DMDC) was purchased from Tokyo Chemical Industry Co., LTD., Tokyo, Japan. GlcNAc, *N*-acetyl-D-glucosamine, was purchased from Nakarai Tesque, Kyoto, Japan. Antibiotics, INH, RFP, EB, streptomycin (SM), and 3-(4,5-dimethylthiazol-2-yl)−2,5-diphenyltetrazolium bromide (MTT) were purchased from Sigma-Aldrich Japan, Tokyo, Japan.

### Bacterial cultures and frozen stock

*M. tuberculosis* H37Rv (ATCC#27294), *Mycobacterium bovis* (ATCC#19210), and *Mycobacterium smegmatis* (ATCC#19420) were purchased from American Type Culture Collection (Manassas, VA, USA). *Mycobacterium avium* 104 was kindly provided by Dr. William Bishai, Johns Hopkins School of Medicine, Baltimore. *M. bovis* strain BCG Tokyo 172 type I (BCG) was kindly provided by Dr. Saburo Yamamoto (National Institute of Infectious Diseases, Japan). *Mycobacterium* bacilli were cultured in Middlebrook 7H9 broth (Difco, Detroit, MI, USA) supplemented with 10% albumin dextrose catalase (ADC) (5% bovine serum albumin [fraction V], 2% dextrose, and 0.004% bovine liver catalase; Difco) and 0.05% Tween 80 and were cultured at 37°C under static conditions. Bacteria were grown to an optical density (OD) of 0.6–0.8 at 530 nm. Then, the cultures were aliquoted and stored at −80°C until needed. The number of colony-forming units (CFUs) in the aliquots was determined by colony assays on Middlebrook 7H11 agar (Difco) supplemented with 10% oleic ADC (OADC) (0.05% oleic acid, 5% bovine serum albumin [fraction V], 2% dextrose, and 0.004% bovine liver catalase; Difco).

*Staphylococcus aureus* clinical isolate was provided by Dr. Imai, the Institute of Public Health, Nagoya City, Japan, and cultured in the heart-infusion broth. *Escherichia coli* strain DH5α was purchased from Takara Bio Inc., Shiga, Japan, and cultured in LB broth. Bacteria were grown to an OD of 0.6–0.8 at 530 nm. Then, the cultures were aliquoted and stored at −80°C until needed. Bacteria were grown to an OD of 0.6–0.8 at 530 nm. Then, the cultures were aliquoted and stored at −80°C until needed.

### *In vitro* broth dilution method to measure minimum inhibitory concentrations of anti-mycobacterial drugs and compounds

The broth dilution for the measurement of minimum inhibitory concentrations (MICs) was carried out according to the method described by Wallace et al. (10). Briefly, 100 µL of the drug solution twofold serially diluted with Middlebrook 7H9 broth medium supplemented with 10% ADC and 0.05% Tween 80 was prepared in a 96-well plate, and then 100 µL of the broth medium containing 1–2 × 10^4^ organisms was added. Then, the plate was incubated at 37°C for 3 days on *M. smegmatis*, for 1 week on *M. avium,* and for 2 weeks on *M. tuberculosis*, *M. bovis,* and *M. bovis* BCG. For *S. aureus* and *E. coli*, the respective media were inoculated 96-well plates in the same manner, and the drugs were added and cultured overnight, after which the turbidity was visually determined.

### Preparation of OCT313-resistant BCG

Ten-fold dilution series of the culture of BCG bacilli, 1.8 × 10^8^ CFU/mL was made with sterilized water, and then 100 µL of the dilution was inoculated onto 7H11 agar supplemented with OADC containing OCT313 at a concentration of 20× MIC. The agar plates were incubated at 37°C for 4 weeks. Colonies were inoculated onto the agar plates containing OCT313 and cultured at 37°C for 4 weeks, respectively. The colonies were subcultured twice on the agar medium containing the compound. Ten colonies were cultured with 7H9 broth supplemented with ADC at 37°C. The bacteria were grown to an OD of 0.6–0.8 at 530 nm, and then the cultures were stored at −80°C until needed.

### Whole-genome analysis of the OCT313-resistant BCG

#### Identification of mutations responsible for drug resistance

A resistant strain *M. bovis* BCG Tokyo 172 was genomically checked to determine mutations responsible for the resistance to OCT313. At first, genomic DNAs were purified from bacilli cultured for 3 weeks on 7H11 agar media according to previous reports (11). Sequencing library was prepared following Kozarewa et al. (12) to avoid PCR bias caused by GC-rich sequence of *M. bovis*. Fragmented genomic DNAs of each strain were conjugated with a tagged sequence. Each of the sheared DNAs was mixed in equimolar quantities and sequenced on Genome Analyzer IIx (Illumina, Inc.) following standard recommended procedures by the manufacturer (Illumina Part #1005361 Rev. C Feb 2010). A parental strain was also analyzed similarly, although HiSeq2000 was used alternatively.

FASTQ files obtained by the sequencing analyses were mapped to a reference sequence (GenBank Accession No. NC_012207) using BWA v0.5.8c. Disagreement nucleotide calls were obtained from the BAM alignment files by Genome Traveler Ver. 1.2 (*In Silico* Biology, Inc.). The specific mutation was determined by the subtraction of the mutations of OCT313 and its parental strain.

### Preparation of recombinant PTA

Genes for PTAs of BCG were amplified by PCR using genomic DNA of BCG Tokyo 172 and its OCT313-resistant strain as a template, and cloned into the pGEM-Teasy plasmid (Promega, Madison, WI) between two *Eco* RI sites. The primers used for PCR were 5′-GGG ATC Ctg gct gac tcc tcg gcg atc ta-3′ (*Bam* HI site underlined) and 5′-GAA GCT TAC TCA TGG ACG CCC TGC GC-3′ (*Hin*d III site underlined). The sequences of the amplified and cloned DNA of PTAs were confirmed by a DNA sequencer (model 3031A; Applied Biosystems, Foster City, CA). The inserted DNA was then recovered and ligated with the expression vector pGEX-5X-1 (GE Healthcare, Piscataway, NJ) using the *Bam* HI/*Eco* RI recognition sites. The *E. coli* strain DH5α was transformed with the resultant constructs, and the fusion protein was induced by culturing at 20°C for 8 h in the presence of 1 mM IPTG. The recombinant GST-PTA fusion proteins were purified using Glutathione Sepharose 4FF (Cytiva) according to the manufacturer’s protocol. Purity of the recombinant protein was more than 95%. Purified proteins were dialyzed against phosphate-buffered saline (PBS) at 4°C. For the preparation of His-tagged PTAs, the insert DNA was then recovered and ligated with the expression vector pQE-32 (Qiagen) using the *Bam* HI/*Hin*d III recognition sites. The *E. coli* strain BL21 was transformed with the resultant constructs, and the His-tagged protein was induced by culturing overnight in auto-induction media (LB was supplemented with 6 g of glycerin, 0.5 g of glucose, and 2 g/L of lactose monohydrate). The recombinant His-tagged proteins were purified using Ni-Sepharose 6FF (Cytiva) according to the manufacturer’s protocol. Purity of the recombinant protein was more than 95%. Purified protein was dialyzed against PBS at 4°C.

### Measurement of the enzymatic activity of PTA

According to the method described by Klotzsch, H.R (13), we measured the enzymatic activity. The principles of measurement of the enzymatic activity are shown below.


$$CoA+Acetyl\;Phosphate\overset{PTA}{⟶}Acetyl-CoA+Pi$$

The activity was measured by a continuous spectrophotometric rate of CoA (A_233 nm_)

Prior to the assay, we prepared the reagents, A: 100 mM Tris HCl buffer, pH 7.4 at 25°C, prepare 100 mL in deionized water using Trizma Base (Sigma No. T-1503), adjust to pH 7.4 at 25°C with 5 M HCl, B: 100 mM glutathione solution (Gluth), freshly prepare 10 mL in reagent A using glutathione, free acid, reduced form (Sigma No. G-4251), C: 6.5 mM coenzyme A solution (CoA), freshly prepare 2 mL in reagent A using coenzyme A, sodium salt (Sigma No. C-3019), D: 220 mM acetyl phosphate solution (Acet Phos), freshly prepare 1 mL in reagent A using acetyl phosphate, lithium potassium salt (Sigma No. A-0262), E: 1 M ammonium sulfate solution ((NH_4_)_2_SO_4_), prepare 50 mL in deionized water using ammonium sulfate (Sigma No. A-5132), F: 25 mM Tris HCl buffer with 500 mM ammonium sulfate, pH 8.0 at 25°C (Enzyme Diluent), prepare 25 mL in deionized water using Trizma Base (Sigma No. T-1503) and ammonium sulfate (Sigma No. A-5132), adjust to pH 8.0 at 25°C with 1 M HCl, G: PTA enzyme solution, immediately before use, prepare a solution containing 1–2 units of PTA in cold reagent F.

The assay procedure was as described below.

Pipette (in milliliters) the following reagents into suitable cuvettes: Reagent A (Buffer), 2.60 (test) or 2.60 (blank); Reagent B (Gluth), 0.05 (test) or 0.05 (blank); Reagent C (CoA), 0.20 (test) or 0.20 (blank); Reagent D (Acet Phos), 0.10 (test) or 0.10 (blank); and Reagent E ((NH_4_)_2_SO_4_), 0.03 (test) or 0.03 (blank).

Mix by inversion and equilibrate to 25°C and monitor the A_233_ until constant, using a suitably thermostatted spectrophotometer, light path = 1 cm. Then add the following (in milliliters): Reagent G (Enzyme Solution), 0.02 (test) or nothing (blank); Reagent F (Enzyme diluent), nothing (test) or 0.02 (blank).

Immediately mix by inversion and record the increase in A_233_ for approximately 5 minutes. Obtain the Δ A_233_ /minute using the maximum linear rate for both the Test and Blank.

The units were calculated using the following formula:


$$Units/mg\;enzyme=(\Delta A_{233}/minTest-\Delta A_{233}/minBlank)\times 3.0\times (df)/(4.44\times 0.02)$$

Units/mgprotein=(units/mLenzyme)/(mgprotein/mLenzyme)

(3.0 = total vol [in milliliters] of assay, df = dilution factor, 4.44 = millimolar extinction coefficient of acetyl-CoA at A_233_ , 0.02 = volume [in milliliter] of enzyme used)

One unit was defined to convert 1.0 µmole of CoA to acetyl-CoA per minute at pH 7.4 at 25°C using acetyl phosphate as substrate. Inhibitors were added to the enzyme solution before the assay. In a 3.0 mL reaction mix, the final concentrations are 98.5 mM Tris, 1.6 mM glutathione, 0.43 mM coenzyme A, 7.23 mM acetyl phosphate, 13.3 mM ammonium sulfate, and 0.02–0.04 unit PTA.

### Overexpression of PTA in OCT313-resistant BCG

The *pta* gene was amplified using Prime STAR HS (Takara Bio Inc.) enzyme with primers (pta-[*Nde* I]-F: 5′-CATATGGCTGACTCCTCGGCGATCTACCTC-3′ and pta-[*Bam*H I]-R: 5′-GGATCCTACTCATGGACGCCCTGCGCCTGAAT-3′), according to the company’s recommended conditions. The PCR products were then cloned into the pTA2 vector (Toyobo Co., Ltd., Osaka, Japan) following the attachment of dA to their 3′ end. The pta-pTA2 was subjected to digestion with *Nde* I and *Bam*H I. The gene fragment was then ligated with the pVV16 (a gift from Professor Crick, Colorado State University), which had been digested with the same restriction enzymes as the pta gene. The BCG Tokyo strain was transformed with the pta-pVV16 by means of electroporation (14).

### Anti-mycobacterial activity under anaerobic environment

This method was described in Wayne et al. (15). All experiments using liquid culture were conducted in Dubos Tween-albumin broth prepared according to the manufacturer’s instructions from Dubos broth base (HiMedia Laboratories No. M067, Pvt. Ltd., Mumbai, India) and Dubos medium albumin (Difco) at a final pH of 6.6 ± 0.2. One milliliter of freeze-stocked BCG (~ × 10^8^ CFU/mL) was added to 100 mL of Dubos medium, and then cultured for 2 weeks at 37°C under static conditions. Twenty-eight milliliters of the bacteria suspension was transferred to 42 mL volume glass tube (Pierce vial No. CV-400, Osaka Chemical, Co., Ltd, Osaka, Japan), containing stir bar (10 mm × 4 mm). After 20 µL of methylene blue solution (1.5 mg/mL) was added to the glass tube, it was cultured at 37°C for 9–14 days with stirring at 130 rpm. After confirming that the culture medium had changed color from blue to yellow, the following experimental procedures were carried out in an anaerobic chamber. The cultured bacterial solution was dispensed in 1 mL portions into the wells of a 24-well plate. The plate was incubated at 37°C for 7 days under anaerobic conditions without stirring, and then 0.01 mL of a solution containing the test compound was added. After further culturing at 37°C for 7 days under anaerobic conditions without stirring, the bacilli were harvested. The bacterial suspensions were diluted with distilled water, and then the appropriately diluted solutions were plated on 7H11 agar supplemented with OADC plates. After the plates were incubated at 37°C for 3 weeks, the colonies on the plate were counted.

### Cell culture and cytotoxicity

The tissue culture medium for human leukemia cell line, THP-1 (acute monocytic leukemia from male), human lung epithelial cell line, A549 (adenocarcinoma cell line derived from male), and human embryonic lung fibroblast cell line, MRC-5 (normal diploid fibroblasts from male) were purchased from JCRB, Tsukuba, Ibaraki, Japan, and were maintained in Dulbecco’s modified Eagle medium (low glucose) with L-glutamine and phenol red (Wako Pure Chemical Industries, Ltd., Osaka, Japan), 100 µg/mL SM (Meiji Seika Pharma Co., Ltd., Tokyo, Japan) and 100 units/mL penicillin G (Meiji Seika Pharma) and 5% heat-inactivated fetal bovine serum (Hyclone, GE Health Life Science, South Logan, UT, USA). The culture was maintained at 37°C in 5% CO_2_ in a 100 mm in diameter Falcon standard tissue culture dish (No. 353003, Thermo Fisher Scientific, Waltham, MA, USA).

Cytotoxicity was measured by measuring the viability of the cells after 3 days with or without the addition of compounds, by staining the cells with crystal violet or the MTT method (16). In adherent cultures, A549 and MRC-5 cells were plated in 96-well flat-bottom microtiter wells, at 1–2 × 10^4^ cells per well in 100 µL of the culture medium, and then 100 µL of the culture medium containing compounds was added. After incubation of the plate for 3 days, the supernatant was removed. The cells were stained with 0.75% crystal violet for 5 min and rinsed with water. Then, 100 µL of 1% SDS was added to each well to dissolve the dye, and the OD at 595 nm was determined using an enzyme-linked immunosorbent assay reader. In the suspended culture cell line, THP-1, proliferation of the cells was determined by MTT assay (16).

### Statistical analysis

The statistical significance of the data sets shown in figures was assessed by one-way analysis of variance with SigmaPlot (Systat Software, Inc., San Jose, CA, USA).

## RESULTS

### Phenotypic and genomic analyses of OCT313-resistant mycobacteria

Colonies of *M. bovis* BCG Tokyo 172 grown in 7H11 agar medium containing OCT313 at a concentration 20× MIC were further passaged into the medium containing the compound. OCT313-resistant bacteria were resistant to OCT313 (Table 1). We have previously reported that the active functional group of OCT313 is DMDC; however, the MIC value of DMDC was four times higher against OCT313-resistant bacteria than against the parent strain, and *N*-acetyl-D-glucosamine (GlcNAc), a sugar backbone of OCT313, alone did not show antibacterial activity against either the parent or the resistant BCG strain (Table 1). The drug susceptibility of OCT313-resistant BCG bacilli against anti-TBTB drugs INH, RFP, SM, and EB was the same as that of the parent strain (Table 1). These results indicate that OCT313-resistant bacteria were resistant only to OCT313.

**TABLE 1:** The antibacterial spectrum of OCT313^*a*^ and interspecies homology of PTA

		*M*. *tuberculosis* H37Rv	*M. bovis*	*M*. *bovis* BCG Tokyo 172	OCT313-resistant BCG	*M*. *avium* 104	*M. smegmatis* mc^2^ 155	*Staphylococcus aureus*	*Escherichia coli* DH5α
*Synthetic compound*									
OCT313		25	31.3	12.5	>200	>100	>100	>100	>100
(GlcNAc-DMDC)									
GlcNAc		>1,000	>1,000	>1,000	>1,000	>1,000	>1,000	>1,000	>1,000
DMDC		1.56	3.13	3.13	12.5	3.91	>100	25	200
*Anti-TB drugs*									
INH		0.04	0.04	0.04	0.04	1.56	3.13	>100	>100
RFP		0.004	0.016	0.01	0.01	0.25	1.56	0.02	12.5
SM		0.39	0.2	0.2	0.2	3.13	0.39	6.25	25
EB		2.5	0.78	3	3	1.6	12.5	>100	>100
*PTA^b^*									
Length	(bp)	2,073	2,073	2,073	2,073	2,089	2,079	987	1,017^*c*^
	(aa)	690	690	690	690	695	692	328	338^*c*^
Homology (%)									
Gene		100	99	99	99	78	74	46	52
Amino acid		100	99	99	99	79	75	46	41

^*a*^ MIC (μg/mL) was measured by the broth dilution method, GlcNAc: *N*-acetyl*-*D-glucosamine, DMDC: dimethyldithiocarbamate, INH: isoniazid, RFP: rifampicin, SM: streptomycin, EB: ethambutol.

^*b*^ The sources of the PTA gene and amino acid sequences are listed in the legend of Fig. 2.

^*c*^ Class 1 PTA.

We investigated the frequency of resistance to OCT313 and anti-TB drugs in the parent BCG strain. The frequency of resistance to OCT313 was 4.2 × 10^−7^, and the frequency of resistance to anti-TB drugs was approximately 10^−6^–10^−7^, except for RFP (Table 2). OCT313 is composed of the active functional group, DMDC, and the sugar backbone, *N*-acetyl-D-glucosamine (GlcNAc). The resistance frequency of DMDC was 2.3 × 10^−5^, so binding to GlcNAc reduced the resistance frequency by approximately 200-fold (Table 2). These results indicate that OCT313 is classified as a compound that is less likely to select for resistance compared to some anti-TB drugs.

**TABLE 2:** Frequency of spontaneously generated drug-resistant colonies in *M. bovis* BCG^*a*^

Agent	Frequency
*Anti-TB drugs*	
INH	2.0 × 10^−6^
RFP	5.2 × 10^−9^
SM	3.1 × 10^−7^
EB	4.5 × 10^−6^
KM	6.3 × 10^−6^
*Synthetic compounds*	
OCT313 (GlcNAc-DMDC)	4.2 × 10^−7^
DMDC	2.3 × 10^−5^

^*a*^ RFP: rifampicin, INH: isoniazid, SM: streptomycin, EB: ethambutol, GlcNAc: *N*-acetyl-D-glucosamine, DMDC: dimethyldithiocarbamate.

Next, we extracted genomic DNA from OCT313-resistant bacteria and analyzed their genome sequences using short-read next-generation sequencing technology.

**Fig 1:**
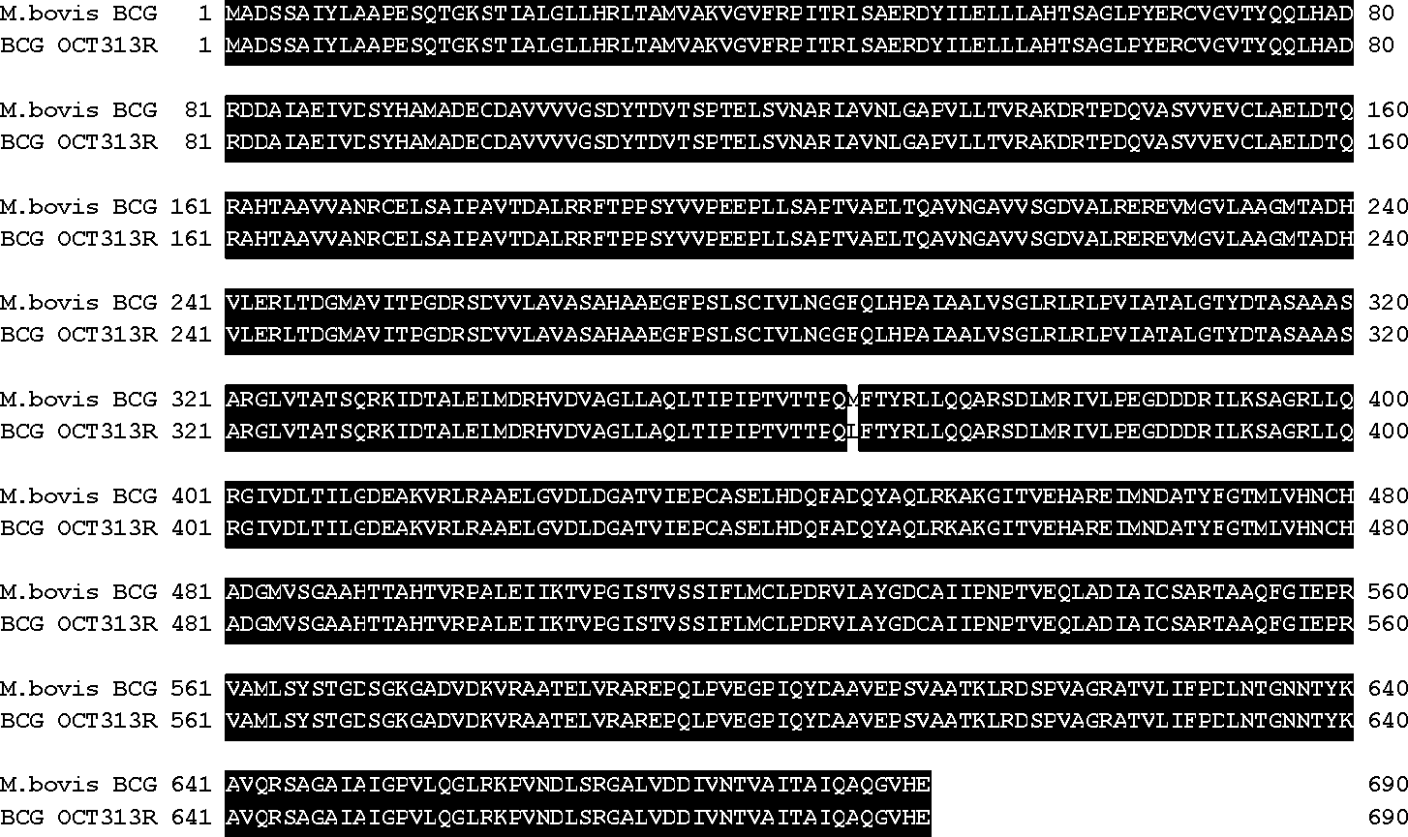
Comparison of the amino acid sequence of the wild-type PTA from *M. bovis* BCG and the mutant PTA (M365L) derived from the OCT313-resistant BCG bacilli. The upper line indicates the amino acid sequence of wild-type PTA, and the lower line indicates the amino acid sequence of mutant PTA (M365L) derived from the OCT313-resistant BCG bacilli. The amino acid residues shown in a black background are conserved in both strains.

The 10 strains of the OCT313-resistant BCG bacilli were selected, and one of them underwent whole-genome sequencing analysis. As a result of single-nucleotide polymorphism analysis, the most frequently mapped gene with known function was a phosphoacetyltransferase (PTA) (EC 2.3.1.8) encoded in the BCG gene JTY_0417. A gene mutation was found in one location compared with the parent BCG Tokyo 172 strain (Fig. 1). This mutation involves a single-base substitution of adenine (A) to cytosine (C) at 1,092 bp, resulting in the conversion of the amino acid methionine to leucine located at amino acid 365 of the protein, composed of 690 amino acids in total (Fig. 1 and Table 1). The results of genome analysis of the OCT313-resistant strain have been registered in the DNA Data Bank of Japan (DDBJ) sequence read archive (#DRA001267), so other mutations can be searched. PTA (M365L) was detected as a mutation involving an amino acid substitution in the bacteria. It was confirmed that all 10 strains, including those whose genomes were analyzed by Sanger sequencing, had the PTA gene mutation (M365L). The MIC values for OCT313 were >100 µg/mL for all 10 strains. We have not examined OCTR_3 and OCTR_4 for abnormalities other than the PTA gene.

**Fig 2:**
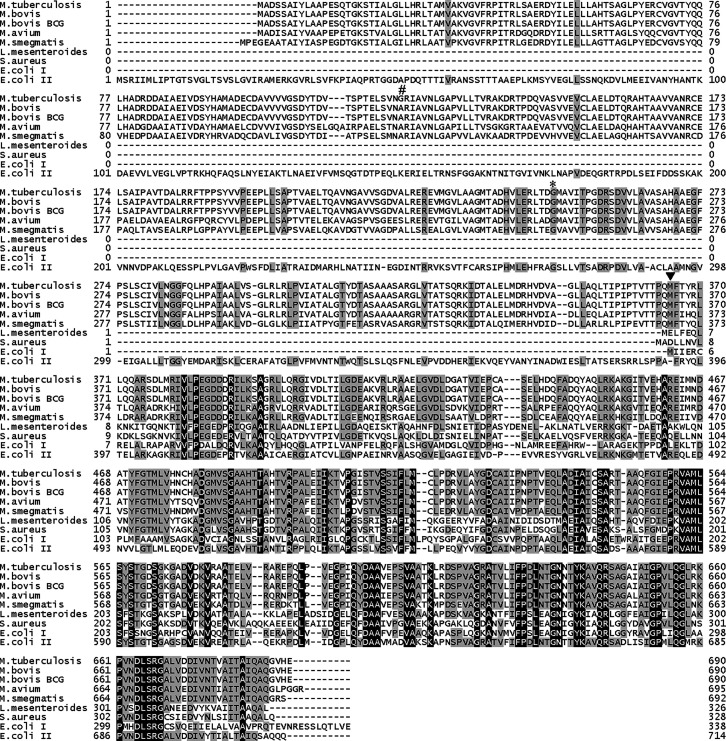
Comparison of amino acid sequences of PTA between *Mycobacterium* and Gram-negative and Gram-positive species. The amino acid sequences of PTA for each bacterial species are shown in the following order from top to bottom: *M. tuberculosis* H37Rv PTA (GenBank #AL123456, Rv0408) (17), *M. bovis* AF2122/97 PTA (GenBank #LT708304, BQ2027_MB0416), *M. bovis* BCG Tokyo172 PTA (GenBank #AP010918, JTY_0417) (18), *M. avium* 104 (GenBank #CP000479, MAV_4760), *M. smegmatis* MC^2^ 155 (GenBank #CP001663, MSMEI_0767), *Leuconostoc mesenteroides* ATCC8293 (NCBI Reference Sequence #NC_008531.1), *S. aureus* DSM 20231 PTA (GenBank #CP104478, N1060_02785), *E. coli* DH5alpha (GenBank #CP026085, C1467_06980) class I PTA (*E. coli* I), and *E. coli* K-12 (NCBI Reference Sequence #NP_416800.1) class II PTA (*E. coli* II). The amino acid sequences shown on a black background are conserved among all bacterial species (100%), and the amino acids shown on a gray background are highly conserved among species (>60%). The solid arrow indicates the mutation site (M365). The asterisk indicates the mutation site in the DRTGG domain in *Salmonella enterica* mutants. # indicates *M. tuberculosis* and *M. bovis* differ by one amino acid residue.

The domain structure of the PTA protein of acid-fast bacteria consists of a regulatory domain in the N-terminus amino acid sequence of approximately 350 amino acids, and a catalytic domain in the C-terminus amino acid sequence of about 330 amino acids, whereas that of *S. aureus* has only a catalytic domain consisting of approximately 330 amino acids (Fig. 2 and Table 1). A mutation in the genome of the OCT313-resistant strain occurred at amino acid 365 in the boundary region between the catalytic and DRTGG domains (Fig. 2). When compared with the amino acid sequences of PTA from other bacterial species, the position of the N-terminal methionine was identical to that of *E. coli* class I PTA (Fig. 2).

There was a correlation between PTA homology among bacterial species and their susceptibility to OCT313 (Table 1). The ineffectiveness of OCT313 against *S. aureus* and *E. coli* may be due to the inability of these PTAs to bind to OCT313.

### Preparation of the wild-type and mutant (M365L) recombinant *M. bovis* BCG PTA enzymes and their inhibitory activity with OCT313

**Fig 3:**
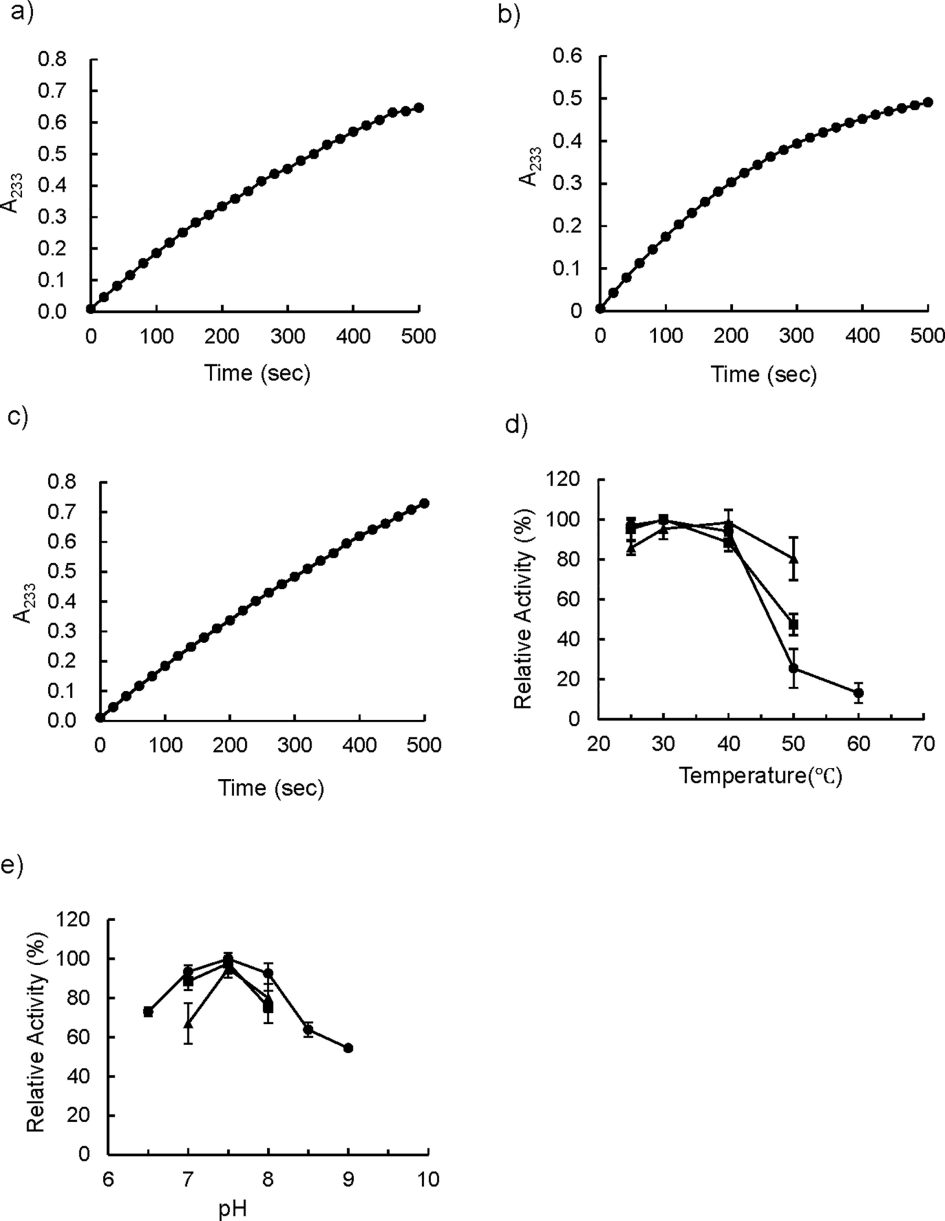
Measurement of enzyme activity of recombinant PTAs. (**a**) The recombinant wild-type *M. bovis* BCG PTA and (**b**) the recombinant mutant PTA(M365L), and (**c**) commercially available *Leuconostoc mesenteroides* PTA. Acetyl-coenzyme A (CoA) formation was monitored by measuring absorbance at 233 nm. PTA activity of the recombinant wild-type BCG PTA, the mutant PTA (M365L), and the *L. mesenteroides* PTA were 18.3, 14.8, and 16.3 U/mg protein, respectively. One unit of activity was defined as the amount of PTA that forms 1 µmol of acetyl-CoA per minute. (**d**) Measurement of the optimum temperature for PTA activity. The relative value of each temperature profile was normalized by the activities at 30°C. (**e**) Determination of optimal pH for PTA activity. The relative value of each pH profile was normalized by the activity at pH 7.5, where the maximum value was obtained. In panels **e** and **d**, circle (●) indicates wild-type recombinant *M. bovis* BCG Tokyo 172 PTA, square (■) indicates the mutant PTA (M365L), and triangle (▲) indicates commercially available *L. mesenteroides* PTA, respectively.

*M. bovis* BCG PTA and mutant PTA (M365L) genes were expressed, respectively, in *E. coli* to obtain a recombinant enzyme. The enzymatic activities of the BCG PTA (12.4 unit/mg protein) and mutant PTA (4.6 unit/mg protein) were comparable with those of a commercially available PTA derived from *Leuconostoc mesenteroides* (16.3 unit/mg protein) (Fig. 3). The optimum temperature and pH of the recombinant PTA enzymes were similar to those of the commercially available *L. mesenteroides* PTA enzyme (Fig. 3d and e).

**Fig 4:**
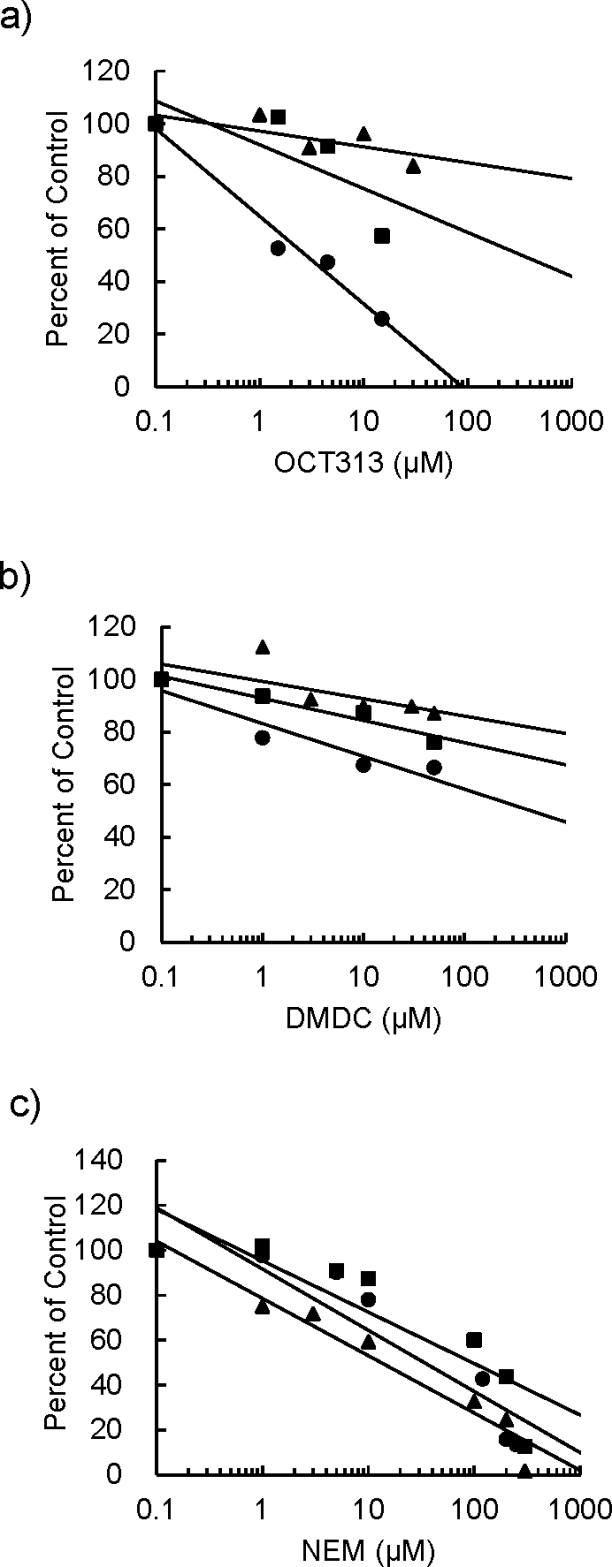
Inhibitory effects of OCT313 on the wild-type PTA and the mutant PTA (M365L) enzyme. The method used to measure PTA enzymatic activity is described in the Materials and Methods section. The inhibitory effect of OCT313 on the wild-type recombinant BCG PTA (rBCG PTA) (●), the mutant rBCG PTA (M365L) (■), and *L. mesenteroides* PTA enzymes (▲) was shown in panel **a**. The inhibitory effects of DMDC on the PTA enzymes are shown in panel **b**. The inhibitory effects of NEM on these PTA enzymes are shown in panel **c**.

Next, we measured the inhibitory activity of OCT313 against the BCG PTA (wild type) and mutant PTA (M365L). The IC_50_ value of OCT313 against wild-type PTA was 2.79 µM, and the value against the mutant was 235.5 µM, which was 84-fold lower (Fig. 4a and Table 3). The IC_50_ values of DMDC for wild-type PTA and mutant PTA were 951 µM and more than 1 mM, respectively, which were almost comparable (Fig. 4b and Table 3). NEM is known to inhibit enzyme activity by binding to cysteine residues and has been reported to inhibit PTA of *Methanosarcina thermophile* (19). NEM inhibited the wild-type and the mutant PTA enzymes at approximately the same concentrations, 14.5 μM and 35.1 µM, respectively (Fig. 4c and Table 3). These results indicate that OCT313 specifically inhibits the wild-type PTA enzyme.

**TABLE 3:** IC_50_ values for the PTA enzymes of OCT313, DMDC, and NEM

PTA enzyme derived from			Inhibitors		
			OCT313 (μM)	DMDC (μM)	NEM (μM)
*M. bovis* BCG^*a*^					
Wild type			2.79	951	14.5
Mutant type (M356L)			235.5	>1,000	35.1
*L. mesenteroides*					
			>1,000	>1,000	50.9

^*a*^ The recombinant PTA enzyme was produced by expressing the PTA gene of *M. bovis* BCG in *E. coli*., DMDC: dimethyldithiocarbamate, NEM: *N*-ethylmaleimide.

**Fig 5:**
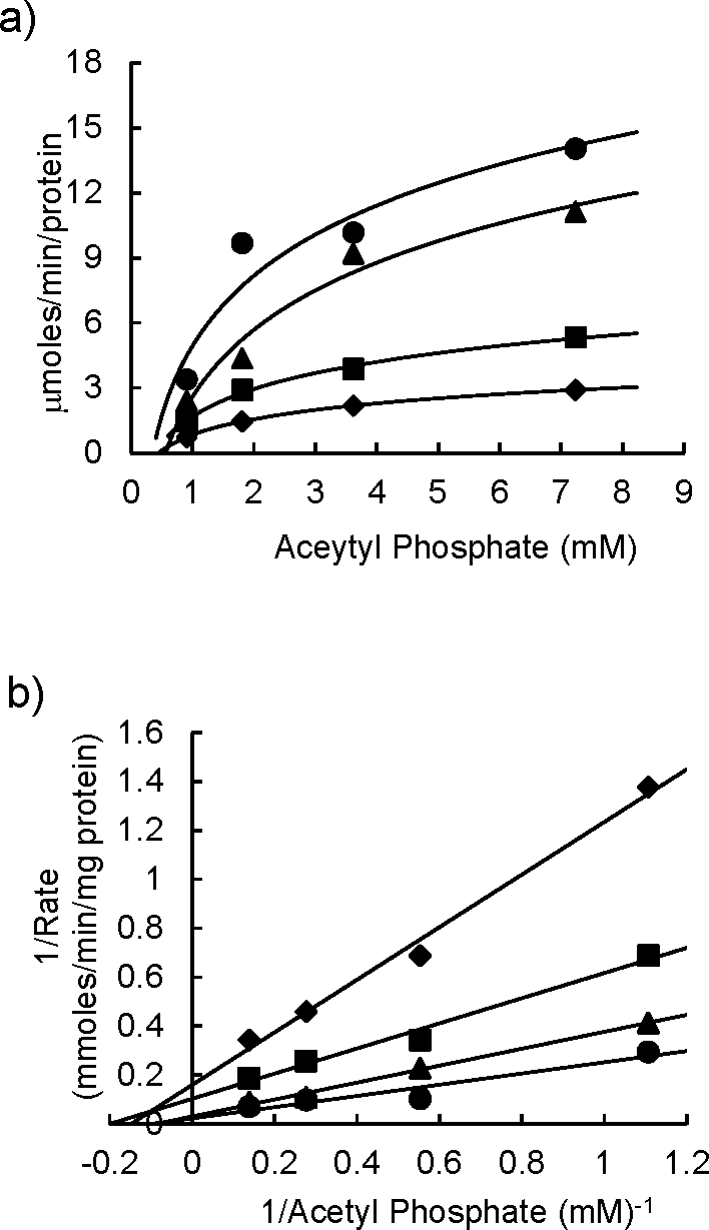
Analysis of inhibition patterns using Lineweaver-Burk plots. The enzymatic activity based on acetyl-CoA formation was measured in the presence of 6.5 mM CoA with varying concentrations of OCT313 using the PTA enzyme assay. Circles, triangles, squares, and diamonds represent OCT313 concentrations of 0, 2.5, 5, and 10 µM, respectively. Panel **a** shows the Michaelis-Menten curve, and panel **b** shows the Lineweaver-Burk plots.

We have previously reported that DMDC is the active functional group in OCT313 with antibacterial activity (7, 8). No difference was observed in the inhibitory effect of DMDC on recombinant wild-type PTA and mutant (M365L) PTA (Table 3). GlcNAc, the sugar structure of OCT313, was not sufficient to inhibit enzyme activity (data not shown). Lineweaver-Burk plots were used to investigate the inhibition of the wild-type PTA by OCT313 to analyze the inhibition pattern. The Km value for acetyl-coenzyme A (CoA) formation by the PTA enzyme was relatively unchanged with increasing concentrations of OCT313, 8.3 ± 2.83 mM, but the Vmax decreased from 43.5 mM to 6.3 mM, suggesting mixed inhibition by OCT313 toward the substrate (Fig. 5).

### Effect of PTA mutations on bacterial growth

**Fig 6:**
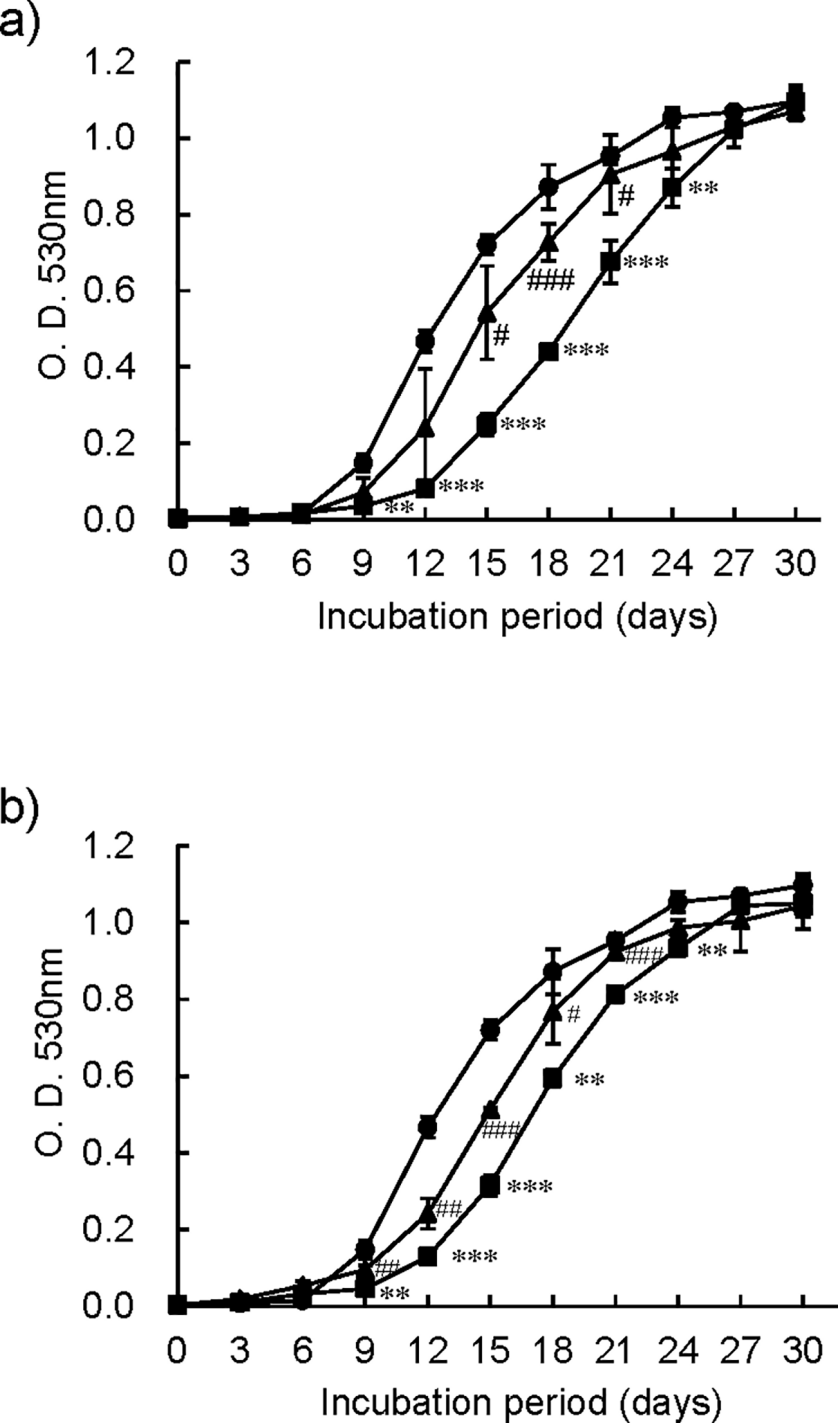
Growth curves of parent strain *M. bovis* BCG Tokyo 172, OCT313-resistant strains, and PTA gene-expressing strains. *M. bovis* BCG Tokyo 172 and OCTR strains, OCTR_3 and OCTR_4, transfected with the pVV16 expression vector, as well as the OCTR strains expressing wild-type PTA, were pre-cultured in 6 mL of Middlebrook 7H9 broth medium supplemented with 10% ADC and 0.05% Tween 80 containing 20 µg/mL kanamycin for several weeks until they reached the stationary phase. The pre-cultured bacterial solution diluted to McFarland No. 1 was further diluted 100 times and cultured in 6 mL of the medium. The bacterial cultures of each strain were carried out in three glass test tubes, and bacterial growth was measured every 3 days using a turbidity meter (mini photometer, model 518R, TITEC Co., Ltd., Nagoya, Japan). Panels **a and b** show the results for OCTR_3 and OCTR_4, respectively. BCG Tokyo 172 transfected with only the pVV16 expression vector, OCTR strains transfected with only the pVV16 expression vector and the strains expressing wild-type PTA are indicated by circles (●), squares (■), and triangles (▲), respectively. The statistical significance of the difference between the bacterial growth of the BCG and the OCTR strains at each time point was analyzed using a one-way analysis of variance, *P < 0.05, **P < 0.01, ***P < 0.001. The statistical significance of the difference between the bacterial growth of the OCTR strains and the OCTR strains expressing wild-type PTA at each time point was analyzed using a one-way analysis of variance, #*P* < 0.05, ##*P* < 0.01, and ###*P* < 0.001.

Next, we investigated the differences in bacterial growth between the OCTR strains transfected with the pVV16 expression vector and the parent strain BCG Tokyo 172 transfected with the vector. Both resistant strains grew slower than the parent BCG strain (Fig. 6). The incubation time to reach half the turbidity of the stationary phase for the BCG, OCTR3, and OCTR4 strains was 12.7 ± 0.2, 19.8 ± 0.4 (*P* < 0.001, vs the BCG), and 17.8 ± 0.5 days (*P* < 0.001, vs the BCG), respectively. When wild-type PTA was expressed in the resistant strains, the time to reach half the turbidity of the stationary phase was significantly shortened to 15.0 ± 0.9 for OCTR_3-wtPTA (*P* < 0.001, vs the OCTR_3) and 15.3 ± 0.8 for OCTR_4-wtPTA (*P* = 0.004, vs the OCTR_4). The OCTR strains grew approximately 1 week slower than the parent strain, indicating that the PTA mutation significantly affected bacterial growth.

### Drug susceptibility of PTA gene-expressed strains in a model of LTBI

Although the model is LTBI, all experiments were performed using *M. bovis* BCG. To determine whether PTA is a drug target of OCT313, we expressed wild-type PTA in OCT313-resistant *M. bovis* BCG Tokyo 172 bacilli and examined their susceptibility to drugs. Furthermore, as PTA is a metabolic enzyme involved in the recycling of acetyl-CoA, which is essential for the tricarboxylic acid (TCA) cycle, it also functions in dormant bacteria. In this study, we investigated the susceptibility of resistant bacteria to OCT313 by overexpressing PTA in the hypoxic Wayne model (15) of LTBI.

**Fig 7:**
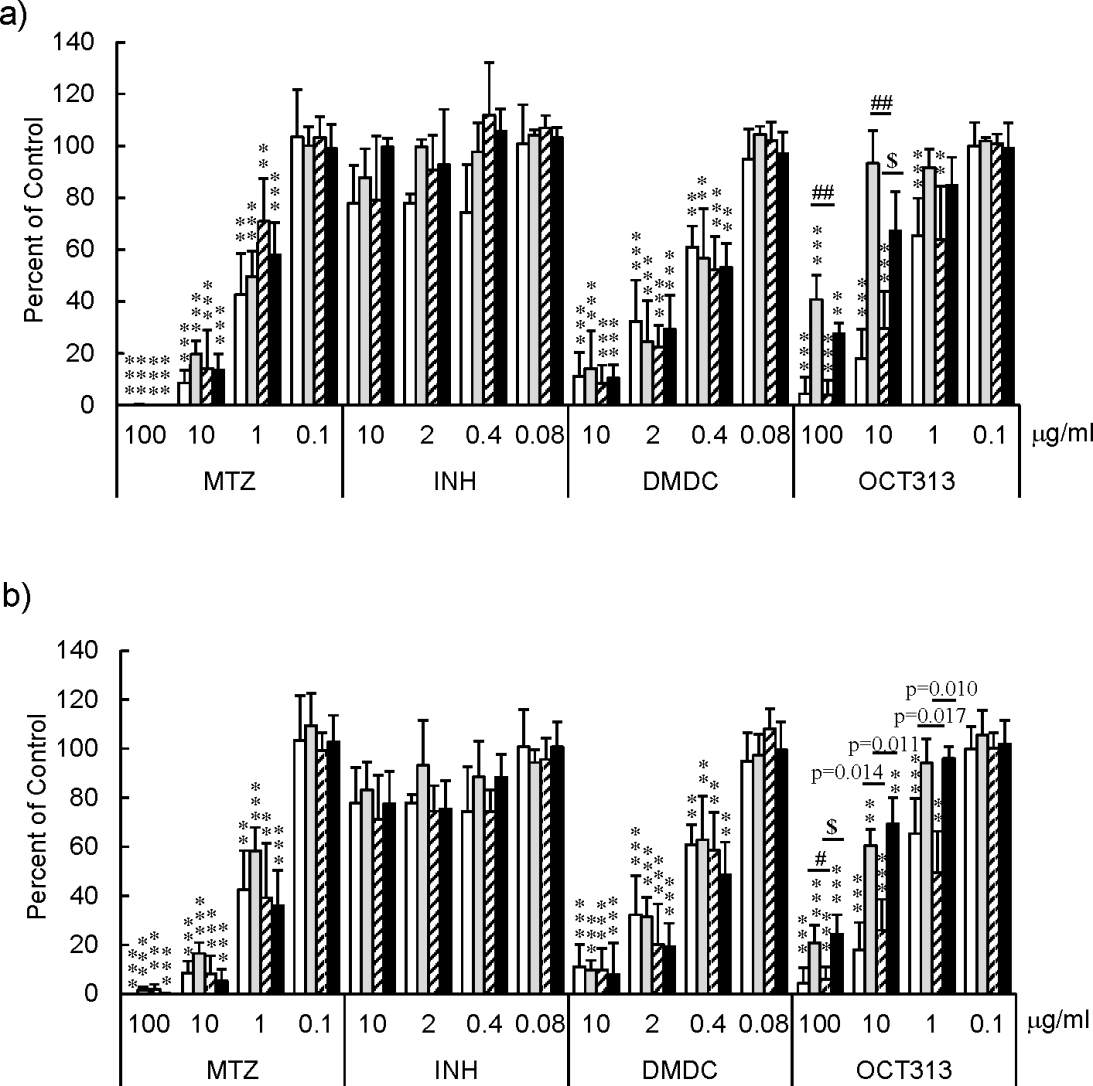
Drug susceptibility of OCT313-resistant strains overexpressing PTA in Wayne dormancy model. The Wayne dormancy model is described in detail in the Materials and Methods section. The x-axis represents the percentage of untreated bacterial colonies. The Y-axis shows the names of MTZ, INH, DMDC, and OCT313. The results for the parent strain *M. bovis* BCG Tokyo172 are indicated by white columns in panels **a and b**. The results for OCT313-resistant strains (OCTR), OCTR_3 and OCTR_4, are shown in panels **a** and **b**, respectively. The gray, hatched, and black columns were OCTR strains transfected with pVV16 expression vector only, the vector integrated into the wild-type PTA gene, and the vector integrated into the mutant type PTA (M365L) gene, respectively. Each experiment was performed in triplicate. The statistical significance of the difference between the result of each experiment and those of the bacilli without drugs was analyzed using a one-way analysis of variance, *P < 0.05, **P < 0.01, ***P < 0.001. Statistically significant differences in sensitivity to OCT313 between the expression vector alone and wild-type PTA-expressing bacteria were also analyzed using a one-way analysis of variance, #*P* < 0.05, ##*P* < 0.01. The statistically significant difference in sensitivity to OCT313 between wild-type PTA-expressing and mutant type PTA (M365L)-expressing bacteria was also analyzed using a one-way analysis of variance, $*P* < 0.05.

As previously reported (15), metronidazole (MTZ) showed antibacterial activity under anaerobic conditions, whereas INH showed reduced activity (Fig. 7).

The parent strain, BCG Tokyo 172, was susceptible to OCT313 in a dose-dependent manner (Fig. 7, white column), while the OCT313-resistant BCG strains, OCTR_3 and OCTR_4, were resistant to OCT313 (Fig. 7a and b, gray column). These results indicate that OCT313 exhibits antibacterial activity against the dormant bacilli and is a potential lead compound for the development of new drugs for the treatment of LTBI. Overexpression of wild-type PTA in the OCTR strains restored their susceptibility to OCT313 (Fig. 7, hatched column). These results indicate that OCT313 has antibacterial activity against dormant *M. bovis* BCG bacteria by inhibiting PTA.

### Cytotoxicity of DMDC and its sugar derivatives, OCT313, against human cell lines

**Fig 8:**
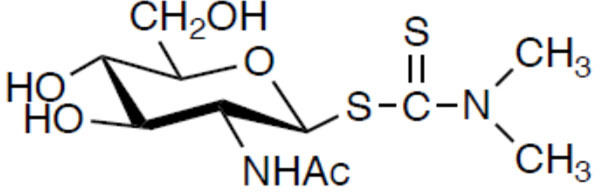
Structure of 2-acetamido-2-deoxy-β-D-glucopyranosyl *N,N*-dimethyldithiocarbamate (OCT313).

In a previous study, we reported that DMDC at the C1 position of GlcNAc in OCT313 (Fig. 8) was active based on activity correlation. We investigated whether the cytotoxicity of DMDC can be reduced by its conversion to a sugar derivative. The LD_50_ values of DMDC against the human-derived cultured cells, THP-1, A549, and MRC-5, were 327, 24, and 11 µg/mL, respectively, whereas those of OCT313 were 8,896, 4,694, and 6,052 µg/mL, which were tens to hundreds times lower (Table 4). The LD_50_ value of OCT313 was comparable to those of other anti-TB drugs. In addition, the safety threshold, which indicates the difference between activity and toxicity, was similar to that of anti-TB drugs, indicating that sugar derivatization increases safety (Table 4). OCT313 is a lead compound with low cytotoxicity.

**TABLE 4:** Cytotoxicity against human cell lines^*a*^

Compound	MIC (μg/mL)	85% survival conc. (μg/mL)			50% lethal conc. LC_50_ (μg/mL)			LC_50_/MIC		
		THP-1	A549	MRC-5	THP-1	A549	MRC-5	THP-1	A549	MRC-5
*Anti-TB drug*										
INH	0.04	100	1,000	250	2,145	3,091	293	53,613	77,277	7,325
RFP	0.004	10	10	10	65	178	65	16,256	44,510	16,250
SM	0.40	100	5,000	1,000	7,163	12,540	1,670	17,908	31,350	4,175
EB	2.5	500	1,000	250	2,291	3,584	278	916	1,434	111
*Synthetic compound*										
OCT313	25	1,250	500	2,500	8,896	4,694	6,052	356	188	242
*Dithiocarbamate*										
DMDC	1.56	200	10	2	327	24	11	210	15	7

^*a*^ THP-1: human leukemia cell line, A549: human lung epithelial cell line, MRC-5: human embryonic lung fibroblast cell line.

## DISCUSSION

In this study, PTA of coenzyme A (CoA) acetyltransferase was suggested as a drug target of OCT313 based on genomic analysis of parental and OCT313-resistant *M. bovis* BCG. The adenine base at position 1,092 of the PTA gene was replaced by a cytosine base in the genome of the OCT313-resistant BCG. Single-nucleotide mutations were detected at the same PTA site in other experimentally generated strains. The frequency of bacterial resistance to OCT313 was low (approximately 10^−8^), compared to other anti-TB drugs, and OCT313 is considered a useful lead compound (Table 2). Notably, sugar derivatization reduced the frequency of resistance by approximately 100-fold compared to the active functional group, DMDC. In addition, the 50% lethal concentrations (LD_50_) values of DMDC against human cell lines THP1, A549, and MRC-5 were reduced by 27-, 196-, and 550-fold, respectively, by sugar derivatization (Table 4). Furthermore, sugar derivatization increased the safety threshold, which indicates the concentration difference between activity and toxicity, by more than 100 times (Table 4).

The mutation in PTA was a non-synonymous mutation in which the amino acid at position 365 of PTA was replaced with leucine instead of methionine (Fig. 1). Analysis of bacteria and archaea revealed the existence of two classes of PTA sequences (20). Class I PTAs are approximately 350 amino acids long, and class II PTAs are twice as long at approximately 700 amino acids (20, 21). The homology of the PTA gene among mycobacterial species, *M. tuberculosis*, *M. bovis*, *M. avium*, and *M. smegmatis,* varies and is even lower in common bacteria such as *S. aureus* and *E. coli* (Fig. 2 and Table 1). Phylogenetic and functional analyses of microbial PTA genes have revealed that class I PTA has only the catalytic domain and class II PTA has two domains, the P-loop and DRTGG, on the N-terminal side of the catalytic domain (22, 23). The domain structure of PTA in *E. coli*. was analyzed in detail. The *E. coli* class II PTA is further composed of three domains conserved in the Conserved Domain Database (CDD) (24): a P-loop containing NTPase at the N-terminal end (CDD cl09099); a DRTGG domain (CDD pfam07085), and a domain shared by phosphate acetyltransferases (CDD cl00390) at the C-terminal end. The DRTGG domain is approximately 120 amino acids long and is located between the P-loop and catalytic domain.

Class II PTAs are further classified into class IIa, comprising both P-loop and DRTGG domain; class IIb, which consists of only the DRTGG domain; and class IIc, which consists of only the P-loop (23). *M. tuberculosis* PTA is classified as class IIb, lacks a P-loop, and has only a DRTGG domain. The DRTGG domain consists of a common amino acid sequence that is conserved in other bacterial species; however, its function is unknown. Functional analysis of truncated class II PTA proteins in *E. coli* demonstrated that the N-terminal regulatory domain is required for maximal enzyme activity and that the N-terminal domain is also involved in the oligomerization of PTA (22). Analysis of a single amino acid mutation in the terminal domain of the acetate metabolism mutant, *acs*, of *Salmonella enterica* showed that the N-terminal regulatory domain is involved in growth at low acetate concentrations (20). The G273D mutation in the DRTGG region of *S. enterica* PTA increased its activity by pyruvate compared to wild-type PTA but did not affect the inhibition by NADH (20). The amino acid G273 in *S. enterica* PTA is conserved in PTAs from *Mycobacteria* and class II *E. coli* PTA (Fig. 2, asterisk).

The homology of the PTA protein differs among bacterial species, and this difference correlates with the antibacterial spectrum of OCT313 (Fig. 2 and Table 1). As the treatment period for mycobacterial infectious diseases, including TB, is longer than that for general antibiotic treatment against common infectious diseases, there is a need to develop narrow-spectrum drugs with fewer side effects, even when taken over a long period. In response to the needs, OCT313 is useful as a lead compound for the development of antibiotics for MDR-TB (7) and LTBI.

Next, we investigated whether OCT313 directly inhibited the enzymatic activity of PTA originating from *M. bovis* BCG, but not *L. mesenteroides.* NEM, a nonspecific inhibitor, inhibited both BCG-derived and *L. mesenteroides*-derived PTA enzymes, whereas OCT313 inhibited only the BCG-derived enzyme, demonstrating that OCT313 specifically inhibits the BCG-derived PTA enzyme (Fig. 4). To examine the specificity of OCT313 for the BCG-derived PTA enzyme, we investigated its inhibitory activity against wild-type and mutant PTA enzymes. The enzyme activity of recombinant wild-type PTA was inhibited by OCT313, but not that of the mutant PTA (M365L) (Fig. 3). Because the mutation site of the OCT313-resistant strain is at the boundary between the DRTGG and catalytic domain, it is expected to affect OCT313’s inhibition of oligomerization or the interaction between the regulatory domain and the catalytic domain (Fig. 1). Lineweaver-Burk plot analysis showed that OCT313 inhibits the activity by the mechanism other than binding to the catalytic domain or competing with the substrate (Fig. 5). Furthermore, the inhibitory activity of the sugar derivative against the PTA enzyme was higher than that of DMDC alone (Fig. 4 and Table 3), suggesting that GlcNAc is an important structural component for the interaction between OCT313 and the PTA enzyme; however, further structural biological analysis, such as crystal structure analysis, is required.

The DMDC did not inhibit PTA (Fig. 4 and Table 3), and the antibacterial activity of the DMDC against these strains was similar between the PTA mutant strains, OCTR_3 and OCTR_4, expressing wild-type PTA and the parent PTA mutant strains (Fig. 7). Therefore, the target of DMDC could be different from that of PTA. Dithiocarbamates chelate metal ions, and the related compound, DSF, exhibits antituberculous activity by chelating copper ions (25). DMDC is the essential functional group of OCT313, but the target of DMDC could be different from that of PTA.

We further investigated whether OCT313 affects PTA in the bacteria. First, we confirmed that the OCT313-resistant strains, OCTR_3 and OCTR_4, grew slower than the parent BCG strain (Fig. 6). Subsequently, we confirmed that the PTA was significantly involved in bacterial growth, as expressing wild-type PTA in these resistant strains restored bacterial growth (Fig. 6).

PTA is involved in the transfer reaction of acetyl groups using CoA as a carrier in metabolic pathways, such as the TCA, in bacteria and human cells. If the target of OCT313 is the metabolic enzyme PTA, it should exhibit antibacterial activity against dormant *M. tuberculosis*. In fact, OCT313 showed antibacterial activity in the Wayne model (15), a model of LTBI (Fig. 7, white column). Moreover, when wild-type PTA was overexpressed in the OCT313-resistant bacterial strains OCTR_3 and OCTR_4, their susceptibility to OCT313 was restored in the model (Fig. 7, hatched column). These results indicate that PTA is a drug target of OCT313. In addition, the fact that OCT313 has a novel site of action is consistent with its antibacterial activity against MDR *M. tuberculosis* (7).

PTA is a critical metabolic enzyme that is widely conserved between prokaryotes and eukaryotes (26). PTA, together with acetate kinase, ACK, and acetyl-CoA synthetase, ACS, plays a role in the production of the central metabolic intermediates, acetyl-CoA and ATP, from acetate (22, 26). The enzymatic reaction of ACS, which produces acetyl-CoA from acetate, is irreversible, whereas those of PTA and ACK, which synthesize acetate from excess acetyl-CoA, are reversible. Acetic acid has been reported to accumulate in granulomas in a guinea pig *M. tuberculosis* infection model (27). Analysis of *M. tuberculosis* mutants lacking ACS showed that the lack of PTA prevented them from growing in the presence of acetate (28). The inhibition of the reversible PTA-ACK pathway is expected to result in excess acetyl-CoA production from acetate via the irreversible ACS pathway. Excess acetyl-CoA activates fatty acid metabolism while inhibiting metabolic pathways that require free CoA (29–31).

Because the homology of PTA genes varies greatly between species (23), it is expected that species-specific PTA inhibitors can be used as selective antibiotics. Recently, *in silico* studies demonstrated that multiple marine-derived natural products associate with PTA of *M. tuberculosis* (32). This study supports the findings by investigating the direct antibacterial activity.

In summary, this study identified PTA as a novel target of OCT313, demonstrating for the first time that inhibition of the metabolic enzyme PTA exerts antibacterial activity against dormant *M. bovis* BCG bacilli. OCT313 is a promising lead compound for the development of treatments against MDR-TB and LTBI.

## AFTER EPUB

*[This article was published on 28 November 2025 with an error in the title. The title was corrected in the current version, posted on 2 December 2025.]*
